# The impaired redox status and activated nuclear factor-erythroid 2-related factor 2/antioxidant response element pathway in wooden breast myopathy in broiler chickens

**DOI:** 10.5713/ajas.19.0953

**Published:** 2020-04-13

**Authors:** Xiaona Pan, Lin Zhang, Tong Xing, Jiaolong Li, Feng Gao

**Affiliations:** 1 College of Animal Science and Technology, Nanjing Agricultural University, Nanjing 210095, China

**Keywords:** Wooden Breast, Meat Quality, Mitochondria, Redox Status, Nrf2/ARE Pathway

## Abstract

**Objective:**

Wooden breast (WB) is a novel myopathy affecting modern broiler chickens, which causes substantial economic losses in the poultry industry. The objective of this study was to evaluate the effect of WB abnormality on meat quality, redox status, as well as the expression of genes of the nuclear factor-erythroid 2-related factor 2 (Nrf2) pathway.

**Methods:**

A total of 80 broilers (Ross 308, 42 days of age, about 2.6 kg body weight) raised at Jiujin farm (Suqian, Jiangsu, China) were used. Twelve unaffected (no detectable hardness of the breast area) and twelve WB-affected (diffuse remarkable hardness in the breast muscle) birds were selected from the commercial broiler farm according to the criteria proposed by previous studies.

**Results:**

The results indicated that WB showed histological lesions characterized by fiber degeneration and fibrosis, along with an increase of muscle fiber diameter (p<0.05). Moreover, higher pH value, lightness, yellowness, drip loss and cooking loss were observed in the WB group (p<0.05). Compared with the normal breast (NOR) group, the WB group showed higher formation of reactive oxygen species (p<0.05), increased level of oxidation products and antioxidant activities (p<0.05), accompanied with mitochondrial damages and lower mitochondrial membrane potential (p<0.05). Meanwhile, the relative mRNA expressions of Nrf2 and its downstream antioxidant genes including heme oxygenase-1, NAD(P)H qui none dehydrogenase 1, glutathione peroxidase, superoxide dismutase, and glutamate-cysteine ligase were higher than those of the NOR group (p<0.05).

**Conclusion:**

In conclusion, WB myopathy impairs meat quality by causing oxidative damages and mitochondrial dysfunction in broilers, even though the activated Nrf2/antioxidant response element pathway provides protection for the birds.

## INTRODUCTION

During the past few decades, the production and consumption of poultry meat continues to increase in the worldwide market. In particular, chicken meat has become one of the most popular muscle foods, owing to its relatively low cost, high nutritive value and suitability for further processing [1]. With the increasing demand for cut-up and further-processed products, as well as consumers’ preference for breast meat, the selection of broilers has been shifted towards rapid growth, high breast-yield, meat conformation and better feed conversion [2]. However, this also results in an increased susceptibility to stress-induced myopathy, such as pale, soft, and exudative-like meat, white striping and wooden breast (WB). The WB is characterized by a remarkable palpatory hardness, out bulging, pale, as well as the occasional presence of white striping and small hemorrhages. Histological observation shows muscle fiber degeneration, inflammatory cell accumulation, and fibrosis [3]. Indeed, the WB condition not only affects the appearance of the *pectoralis major* muscle, but also impairs meat quality and functional properties, leading to huge economic losses in broilers industry [1,4]. Although the degeneration with fibrosis and lipidosis was found in WB tissue, no health hazard is related to the consumption of WB meat [5].

Up till now, several studies carried out to detect differential genes, proteins and metabolites related to the occurrence of WB have evidenced a complex etiology of this defect [6–8]. However, the exact mechanisms in the development of WB are not fully understood. Thus, a clear understanding on the underlying causes and mechanisms are needed. Recent studies reported that hypoxia is recognized as the most likely cause of WB in broilers [8,9]. Additionally, the increased fiber size in fast-growing broilers often accompanies lower capillarity density, which reduces oxygen supply to muscle and further contributes to the oxidative stress [3,8]. This hypothesis is supported by histological observations that the muscle lesion severity gradually decreases from the ventral side towards the dorsal section of the *pectoralis major* muscle [10]. Therefore, oxidative stress is becoming a major concern for the development of WB. Oxidative stress is considered as an event of increased formation of free radicals in the cell. In particular, the excessive reactive oxygen species (ROS) may lead to oxidative damage through attacking almost all the cellular macromolecules, which affect normal metabolism of animals [11]. Generally, mitochondria respiration is the main source of ROS, and the mitochondria are the primary target attacked by ROS. Thus, the excessive ROS may result in irreversible damages of mitochondria [12].

The excessive production of ROS can stimulate the antioxidant defense system, of which, the nuclear factor-erythroid 2-related factor 2 (Nrf2) pathway has been identified as an important endogenous mechanism for suppressing oxidative stress [13]. The Nrf2 pathway plays an important role in preventing and reducing oxidative injuries via up-regulating antioxidant enzymes responsible for the detoxification of ROS in broilers [14]. Nrf2 is a transcription factor, which possesses an antioxidant response element (ARE) in the promoter region and regulates the expression of various cytoprotective enzymes [15]. Under normal conditions, Nrf2 is sequestered and constantly ubiquitinated by Kelchlike ECH-associated protein-1 (Keap-1) in the cytoplasm. Under oxidative activation, Nrf2 binds to the ARE and induces the expression of target genes [16].

Yet, the high incidence of WB myopathy leads to an increasing concern for producers and consumers. However, to our knowledge, limited studies have been conducted concerning the redox status in WB abnormality. Therefore, the purpose of this study was to investigate the oxidative status of broilers based on the categorized normal and WB samples, and to evaluate the potential protective roles of Nrf2/ARE pathway in alleviating this myopathy, which can provide a better understanding of the relationship between redox status and meat quality changes in WB abnormality.

## MATERIALS AND METHODS

### Ethical statement

All experimental procedures were approved by the Institutional Animal Care and Use Committee of Nanjing Agricultural University (GB14925, NJAU-CAST-2011-093), and the serial number of the laboratory animal use certificate issued by Science and Technology department of Jiangsu province is SYXK (Su) 2017–0007.

### Animals and rearing management

For this study, a total of 80 broilers (42-day-old Ross 308, about 2.6 kg body weight) raised at Jiujin farm (Suqian, Jiangsu, China) were used. The broilers were raised on a basal diet formulated according to the NRC (1994) nutrient requirements for 1 to 21 days starter diet and 22 to 42 days grower diet. The composition and nutrition levels of the basal diet are given in Supplementary Table S1. Chickens were provided with commercial standard management, the temperature was maintained at 32°C to 34°C from day 1 to 3, then gradually reduced to 22°C at the rate of 2°C to 3°C per week. Birds were allowed free access to feed and water toward the end of rearing.

### Animal selection and sample collection

According to the criteria proposed by previous studies, broilers were separated depending on visual observations for posture and manual palpation of the breast muscle in a cranio-caudal direction: unaffected chickens are able to lift wings easily and no detectable hardness of the breast area; WB-affected chickens exhibit bent-forward posture and are unable to lift their wings sufficiently, breast area being markedly firm on palpation [17,18]. Broilers were slaughtered by cervical dislocation and exsanguination, and a postmortem examination was performed immediately. Consistent with the criteria proposed by Sihvo et al [3] and Papah et al [18], a trained and skilled operator eventually selected twelve WB fillets (diffuse remarkable hardness in the breast muscle, especially in the cranial area) and twelve normal breast (no detectable hardness area in the breast muscle) by palpation of the *pectoralis major* muscle.

After selection, the entire right breast muscle was collected, placed into polyethylene bags and stored at 4°C for the measurement of meat quality. A cranial fraction of the left *pectoralis major* muscle was cut to 1 mm×1 mm×1 mm shapes and stored in 2.5% glutaraldehyde phosphate buffer saline (v/v, pH 7.2) immediately for the ultrastructural observation. At the same location, approximately 0.5 cm wide by 0.5 cm deep by 3 cm long histological samples parallel to the muscle fibers were removed, and fixed in 10% formalin buffer. A piece of *pectoralis major* muscle tissue was rapidly treated for the detection of the level of ROS and the isolation of mitochondria. Within 20 min postmortem, 10 g of the cranial of the left pectorals major muscle was obtained and frozen in liquid nitrogen, and then stored in −80°C for biochemical tests.

### Histologic examination

An examination of the microscopic structure was conducted to ensure the selected samples exhibited typical characteristics of normal and WB. Histological evaluations were performed using the method of Clark and Velleman [10] with minor modifications. Muscle samples were stored in 10% formalin buffer for 24 h at room temperature. Specimens were oriented transversally, dehydrated in a graded series of ethanol and embedded in paraffin. From each sample, consecutive sections (6 μm thick) were obtained and stained with hematoxylin and eosin. Assessment of myopathic lesions of each slides was performed using a light microscope (Olympus Corporation, Tokyo, Japan). Muscle fiber diameter was determined according to the method of Kawasaki et al [19] with some modifications. The vision was randomly selected, 15 fibers around the arbitrary fiber were measured and repeated 10 times per sample using Image-Pro Plus software.

### Measurement of meat quality

At 24 h postmortem, the ultimate pH of the *pectoralis major* was evaluated using a HI9125 portable waterproof pH/ORP meter (HANNA Instrument, Padova, Italy) according to the method of Brambila et al [20] Individual fillets was measured three times at the cranial locations, the values were averaged.

Meat color (CIE L* a* b*) was measured on the dorsal surface of each sample using a CR410 Chroma Meter (Konica Minolta Sensing Inc., Osaka, Japan). All samples were measured in triplicate, and mean values were calculated.

Approximately 20 g of each fillet was cut into a regular shape and weighed (*W*_1_), then suspended on a hook and placed in sealed container, stored at 4°C. The samples were reweighed (*W*_2_) 24 h later. The drip loss was calculated:

$$\text{Drip loss rate }(\%)=\frac{W_{1}-W_{2}}{W_{1}}\times 100\%$$

At 24 h post mortem, about 30 g specific-shaped tissue of each fillet was obtained for the cooking-loss determination. The samples were weighed (*W*_3_) and packed in a ziplock bag, then cooked at 80°C in a water bath until the internal temperature reached 70°C. The samples were weighed (*W*_4_) after cooking. The cooking loss was calculated:

$$\text{Cooking loss rate }(\%)=\frac{W_{3}-W_{4}}{W_{3}}\times 100\%$$

As to shear-force, the cooked samples were cut into two strips (1 cm×1 cm×3 cm) along the direction of the muscle fibers. Then, each strip was measured three times using a Digital Meat Tenderness Meter (Model C1LM3, Northeast Agricultural University, Harbin, China), and average values were calculated.

### Detection of reactive oxygen species

The oxidation of 2′7′-dichlorodihydrofluorescein diacetate level was determined as the index of ROS produced by cellular components, using a commercial kit (E004, Nanjing Jiancheng Bioengineering Institute, Nanjing, China), according to the manufacturer’s instructions.

### Mitochondrial membrane potential measurement

The fresh muscles were treated for the isolation of mitochondria by using a commercial kit (C3601, Beyotime Biotechnology, Shanghai, China). Mitochondrial membrane potential (MMP) assay kit with JC-1 (C2006, Beyotime Biotechnology, China) was used to detect the MMP changes of each breast samples, in accordance with the manufacturer’s instructions.

### Ultrastructural observation

Specimens were washed twice with phosphate buffer and then fixed in 1% osmium tetroxide solution (v/v). After dehydrated using acetone and embedded in Epon 812, ultrathin (≤90 nm) sections were cut, and then stained with uranyl acetate and lead citrate. The alterations in mitochondrial morphology were observed with a transmission electron microscope (HT7700, Hitachi, Tokyo, Japan).

### Measurement of oxidative parameters

Muscle tissues were homogenized in chilled phosphate saline buffer, and then centrifuged at a speed of 1,000 rpm for 15 min at 4°C. The supernatant was obtained for the assessment of oxidative parameters. The measurements of total antioxidant capacity (T-AOC), superoxide dismutase (SOD), glutathione peroxidase (GPX), protein carbonyl, malondialdehyde (MDA), and lipid peroxidation (LPO) were performed using commercial kits (Nanjing Jiancheng Bioengineering Institute, China), in accordance with the manufacturer’s instructions. The 8-hydroxydeoxyguanosine (8-OHdG) was measured using a commercial enzyme-linked immunosorbent assay kit (Angle gene Bioengineering Institute, Nanjing, China) per the manufacturer’s instructions. The protein concentration was measured using the Coomassie brilliant blue method by a protein assay kit (Nanjing Jiancheng Bioengineering Institute, China) for standardization.

### RNA extraction and gene expression analysis

Total RNA from the breast muscle samples was isolated using RNAiso Plus reagent (Takara Biotechnology Co. Ltd, Dalian, China). After measuring the purity and quality, reverse transcription was performed using PrimeScript RT Master Mix (Takara Biotechnology Co. Ltd., China). The real-time polymerase chain reaction (PCR) was performed on an ABI PRISM 7500 Detection System (Applied Biosystems, Foster City, CA, USA) with the reaction protocol as follows: one cycle at 95°C for 30 s; 40 cycles at 95°C for 5 s, and 60°C for 30 s. Primer sequences used for real-time PCR are shown in Table 1. The relative amount of target gene mRNA was calculated using a 2^−ΔΔCt^ method [21]. Primer sequences are shown in Table 1.

### Statistical analysis

The statistical analysis of the data was performed by one-way analysis of variance with software SPSS Statistics 20.0 (SPSS Inc, Chicago, IL, USA). The results were presented as mean values and standard error of the means. Significant difference was defined as p<0.05.

## RESULTS

### Histological analysis

**Figure 1:**
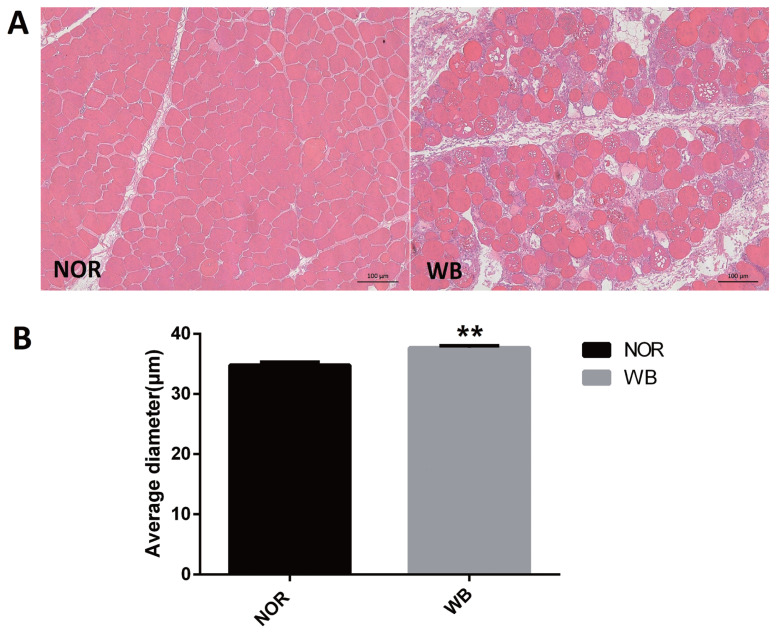
Histological observations (A) and average diameter (μm) of muscle fiber (B) of normal (NOR) and wooden breast (WB) *pectoralis major* muscle of broilers. Scale bars: 200 μm. The results are expressed as the means±standard error (n = 12). ** Shows highly significant difference (p<0.01) compared with the NOR group.

As shown in Figure 1A, the normal breast (NOR) samples exhibited well-organized myofibers with characteristic polygonal shape. In contrast, multifocal degeneration and fibrosis were observed in the WB tissues. In addition, the necrotic region was characterized by diffuse collagen deposition and occasional infiltration by inflammatory cells. In addition, a wide variation in fiber size was also detected in WB-affected samples. The degenerative lesions were accompanied by giant and hypertrophic fibers. The histological analysis showed that the average diameter of myofibers of the WB group was higher than that of the NOR group (p<0.05, Figure 1B).

### Meat quality

The basic indicators of meat quality are shown in Table 2. Compared with the NOR group, the WB group exhibited higher pH, lightness, and yellowness values (p<0.05). Moreover, significant differences were observed between the NOR and WB groups for drip loss and cooking loss (p<0.05). There were no significant differences in the redness and shear force.

### Reactive oxygen species level and redox status

The antioxidant capacities are presented in Table 3. The production of ROS was significantly higher in the WB group compared to the NOR group (p<0.05). The WB group exhibited significantly higher activities of T-AOC, SOD, and GPX (p<0.05). Additionally, the contents of protein carbonyl, MDA, LPO, and 8-OHdG were significantly higher in the WB group than those of the NOR group (p<0.05).

### Ultrastructural alterations and mitochondrial membrane potential

**Figure 2:**
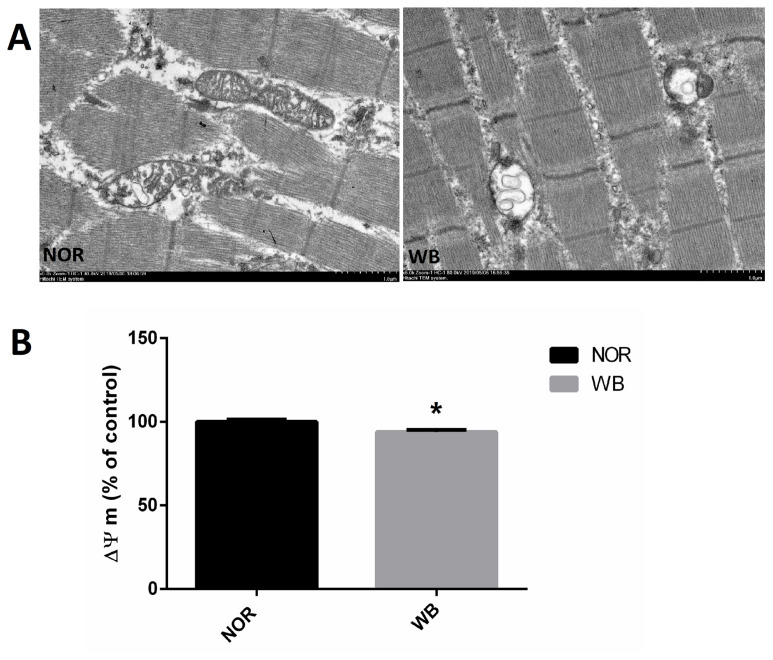
Transmission electron micrographs (A) and mitochondrial membrane potential (B) of *pectoralis major* muscle of broilers. NOR, normal breast; WB, wooden breast. The results are expressed as the means±standard error (n = 12). Bar: 1 μm. * Shows significant difference (p<0.05) compared with NOR group.

As shown in Figure 2A, well-developed mitochondria with membrane integrity and rich cristae were found in the NOR group. In contrast, irregular-shaped mitochondria were observed in the WB samples, exhibiting disrupted and poorly defined cristae in the swollen mitochondria. In addition, compared to the NOR group, the values of MMP were lower in the WB group (p<0.05, Figure 2B).

### Gene expressions

**Figure 3:**
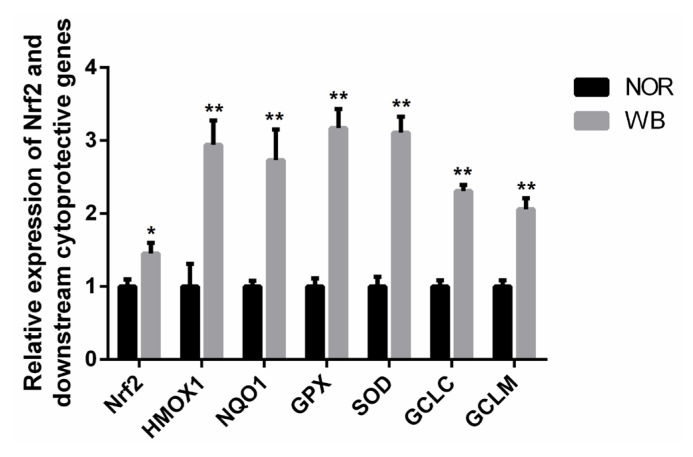
Relative mRNA expressions of *Nrf2* and its downstream antioxidant genes of *pectoralis major* muscles from normal (NOR) and wooden breast (WB) of broilers. *Nrf2*, nuclear factor erythroid 2-related factor 2; *HMOX1*, heme oxygennase 1; *NQO1*, NAD(P)H quinone dehydrogenase 1; *SOD*, superoxide dismutase; *GPX*, glutathione peroxidase; *GCLC*, glutamate cysteine ligase catalytic; *GCLM*, glutamate cysteine ligase modifier. Values are means±standard error, n = 12. *, ** Show significant difference (p<0.05) and highly significant difference (p<0.01) compared with NOR group respectively.

As shown in Figure 3, the WB-affected birds exhibited a significantly higher relative mRNA expression of Nrf2. In addition, mRNA expressions of its downstream target genes including heme oxygenase-1 (*HMOX1*), NAD(P)H qui none dehydrogenase 1 (*NQO1*), *GPX*, *SOD*, glutamate cysteine ligase catalytic subunit (*GCLC*), glutamate cysteine ligase modifier subunit (*GCLM*) were significantly higher in the WB group than those of the NOR group (p<0.05).

## DISCUSSION

In this study, WB-affected broilers were selected based on the observation of their inability to lift wings and manual palpation. In addition, microscopic observations were further conducted to verify the accuracy of sample selection. All considered WB samples exhibited mild to severe structural abnormalities such as round-shape fibers, muscle fiber hyalinization, nuclear internalization and interstitial thickening with fibrosis as reported by Sihvo et al [3]. Moreover, the presence of irregular muscle fiber was observed in WB, including giant-type fibers and occasional small caliber myofibers. These results were in agreement with previous studies [3,5,22]. Muscle fiber hypertrophy is associated with genetic pressure for rapid growth of breast muscle [23]. In our present study, the average myofiber diameter in the WB group was higher than that of the NOR group, which is in agreement with previous studies [11,22,24]. The increased large myofibers outgrow their life support systems ultimately, resulting in myodegeneration [2]. Furthermore, the larger size of fibers in the *pectoralis major* muscle decreases the available space for vascularization and therefore triggers hypoxia, which might contribute to oxidative stress and, subsequently, affect muscle development and structure.

Damages to the muscle morphological structure ultimately influence meat quality, since meat quality attributes are closely related to the histological structure of muscle tissue [25]. With respect to pH, several previous studies indicated that the higher ultimate pH is ascribed to a decreased muscle glycogen content and an altered glucose metabolism [7,8]. Muscle fibers affected by severe fibrosis are usually replaced by connective tissues, and there is extensive collagen cross-linking in the case of WB [2]. Muscles with higher collagen content exhibit harder texture, which might explain the palpatory hardness in WB conditions [4,26]. However, as for cooked meat, there was no difference in shear force between the WB and NOR samples in this experiment. Several studies have reported similar results that WB does not show a significant influence on shear force [22,26]. In this regard, the denatured structural proteins of the muscle may account for the comparable shear force with normal breast [27]. Moreover, poor cohesion and increased muscle fiber shrinkage during cooking might affect texture characteristics of WB meat [22]. The higher drip loss and cooking loss indicated the reduction of water holding capacity (WHC), which is mainly related to the degeneration of muscle tissue [3–5]. Furthermore, the presence of a thin layer of fluid viscous material on the WB surface due to myodegeneration increased natural light scattering, resulting in the pale syndrome of WB meat. These outcomes are in agreement with previous findings [4,22].

The primary source of ROS in skeletal muscle is the leakage of electrons from the respiratory chain in mitochondria. Meanwhile, mitochondria are vulnerable to attack by ROS owing to the lipid (phospholipid) and protein composition of their membranes. In the current study, a higher level of ROS in WB samples indicated an excessive formation of free radicals, which was in agreement with the findings reported by Salles et al [28] in white striping myopathy. In particular, the disruption of mitochondria structure observed in the ultrastructure observation might be caused by the oxidative injury of phospholipids, which consequently accelerates the oxidation process and promote further ROS generation [29]. Furthermore, WB muscles showed lower MMP, which might be related to mitochondrial dysfunction as the MMP has been shown to be sensitive to lipid peroxidation [30]. These results suggested that the overproduction of ROS in WB-affected birds leads to damages of mitochondrial structure and causes mitochondrial dysfunction, which subsequently enhances ROS generation.

In broiler chickens, excessive production of ROS in skeletal muscle causes cellular damage by leading to protein oxidation, lipid peroxidation and DNA modification [11]. According to our results, higher contents of protein carbonyl, MDA, LPO, and 8-OHdG were observed in WB samples, which indicated that oxidative stress occurs in WB-affected birds. Similarly, Soglia et al [5] reported the disturbed redox status in WB conditions, including the oxidation of muscle proteins and lipids. Muscle cells are prone to oxidative stress because of the high content of phospholipids in the membranes. Therefore, the excessive production of ROS in WB muscles might cause damage to the cell membrane, impair cell integrity and lead to muscle dysfunction. The protein carbonyl is a representative biomarker of oxidative damage to muscle proteins. According to our results, the higher level of protein carbonyls suggested a widespread oxidation of proteins in WB conditions. It is reported that ROS attacks myofibrillar proteins during maturation and storage [31], this might explain the reduction of WHC as it is mainly dependent on myofibrillar proteins. Thus, the extensive loss of membrane integrity and the oxidation of proteins in WB might contribute to the impaired meat quality traits [3,5]. DNA damage caused by oxidative stress can lead to aging and cancer, however, its impact on meat quality is limited [32]. Therefore, oxidative stress is the main cause of the initiation and progression of deterioration of meat quality in WB-affected broilers.

Under stressful conditions, excessive free radicals can break down the balance between pro-oxidant and antioxidant systems. Traditionally, it is considered that the excessive ROS can be removed or reduced by antioxidant enzymes such as GPX and SOD. The enhanced ROS generation can lead to oxidative damages and decrease antioxidant enzymes activities [12]. However, in our present study, the WB group showed significantly increased activities of T-AOC, GPX and SOD compared with the NOR group, indicating the antioxidant enzyme defensive system might be activated in WB-affected broilers in order to increase the scavenging capacity of free radicals, which further reduces the oxidative damage to breast muscle. Similarly, Salles et al [28] reported that broiler breast fillets with severe white striping myopathy exhibit excessive production of free radicals, in concert with the elevated levels of antioxidant responses. In addition, Sohail et al [33] found an increase in total antioxidants in the heat stressed broilers, which is considered a protective response against oxidative damage.

Nrf2 is a key transcription factor protecting cells against oxidative damage, and is considered as a molecular switch in targeting of ARE-mediated expression of antioxidant genes [15]. In the present study, the mRNA expression of Nrf2 in the WB group was significantly higher than that of the NOR group, suggesting that there might be more Nrf2 molecules translocated into the nucleus. Subsequently, the activated Nrf2 induced the transcription of several downstream cytoprotective enzymes. Based on the increased oxidation status observed in WB muscles, the higher expression of Nrf2 implied that the Nrf2/ARE pathway was activated in response to oxidative stress. Xu et al [14] reported that the MAPK/Nrf2/ARE antioxidant signaling pathway can be activated in order to overcome the oxidative stress stimulated by low-current & high-frequency electrical stunning in broilers. Under the stimuli of oxidative stress, Nrf2 dissociates from Keap-1, and translocates into the nucleus and binds to the ARE in the promoter of anti-oxidative genes, such as *HMOX-1*, *NQO1*, *SOD*, *GPX* and glutamate cysteine ligase, which protect cells from oxidative damages [15]. In the present study, we found that the mRNA expressions of these anti-oxidative genes in the WB group were significantly higher than those of the NOR group, which was consistent with the results of the antioxidant enzymes, confirming that the activated Nrf2 increases the levels of its downstream antioxidative genes so as to alleviative the oxidative status. Overall, we speculate that the activation of Nrf2/ARE pathway and the induction of antioxidative genes could promote the restoration of the balance between oxidants and antioxidants in WB-affected birds.

In conclusion, the occurrence of WB myopathy significantly affects muscle structure and meat quality, which are related to the destroyed cellular redox status. Thus, besides clinical evaluations and histologic examinations, analysis of the cellular redox homeostasis is also important for WB determination.

## Figures and Tables

**Table 1:** Primer sequences used for real-time polymerase chain reaction

Genes	Primer sequence (5′ → 3′)	Product size (bp)	Gene bank number
*Nrf2*	F: CAGGCCGTCTTGAAGCTCATCTC	179	NM_205117.1
	R: CTTGCCTCTCCTGCGTATATCTCG		
*HMOX1*	F: ACGTCGTTGGCAAGAAGCATCC	181	NM_205344.1
	R: TTGAACTTGGTGGCGTTGGAGAC		
*NQO1*	F: TCGCCGAGCAGAAGAAGATTGAAG	191	NM_001277621.1
	R: GGTGGTGAGTGACAGCATGGC		
*SOD*	F: GGTGACCTCGGCAATGTGACTG	93	NM_205064.1
	R: AATGATGCAGTGTGGTCCGGTAAG		
*GPX*	F: AAGTGCTGCTGGTGGTCAACG	155	NM_001277853.2
	R: GTTGGTGGCGTTCTCCTGGTG		
*GCLC*	F: GAAGCACGGCATCCTCCAGTTC	151	XM_419910.5
	R: TTCTTCGCCACTCAGCACTAAGC		
*GCLM*	F: GCCATAGGCACCTCTGACCTTG	111	NM_001007953.1
	R: CGGCATCACGCAACATGAAGC		
*GAPDH*	F: GGTAGTGAAGGCTGCTGCTGATG	200	NM_204305.1
	R: AGTCCACAACACGGTTGCTGTATC		

*Nrf2*, nuclear factor erythroid 2-related factor 2; *HMOX1*, heme oxygennase 1; *NQO1*, NAD(P)H quinone dehydrogenase 1; *SOD*, superoxide dismutase; *GPX*, glutathione peroxidase; *GCLC*, glutamate cysteine ligase catalytic subunit; *GCLM*, glutamate cysteine ligase modifier subunit; *GAPDH*, glyceraldehyde-3-phosphate dehydrogenase.

**Table 2:** Analysis of meat quality of normal and wooden breast *pectoralis major* muscle of broilers

Items	Category1)		SEM	p-value

	NOR	WB		
pH_24 h_2)	5.87	6.08**	0.033	<0.01
Lightness (L*)	49.86	53.33**	0.463	<0.01
Redness (a*)	1.22	1.03	0.071	0.170
Yellowness (b*)	1.88	3.93**	0.288	<0.01
Drip loss (%)	1.70	2.32**	0.001	<0.01
Cooking loss (%)	17.33	22.86**	0.007	<0.01
Shear force (N)	24.25	22.60	0.652	0.218

SEM, standard error of the means.

1) NOR, normal breast; WB, wooden breast. Data are the mean of twelve replicates (n = 12).

2) pH_24 h_, pH at 24 h post-mortem.

** Represents highly significant difference (p<0.01) compared with the NOR group respectively.

**Table 3:** Reactive oxygen species production, antioxidants activity and content of oxidative products of normal and wooden breast *pectoralis major* muscle of broilers

Items	Category1)		SEM	p-value

	NOR	WB		
ROS generation (% of NOR)	100	115**	2.689	<0.01
T-AOC (mmol/g of protein)	0.034	0.047*	0.003	0.026
SOD (U/mg of protein)	48.43	56.34**	1.396	<0.01
GPX (U/mg of protein)	6.62	10.15**	0.545	<0.01
Protein carbonyl (nmol/mg of protein)	2.07	2.96*	0.224	0.041
MDA (nmol/mg of protein)	0.33	0.75**	0.071	<0.01
LPO (μmol/mg of protein)	0.12	0.26**	0.023	<0.01
8-OHdG (ng/g of protein)	0.55	0.64*	0.021	0.012

SEM, standard error of the means; ROS, reactive oxygen species; T-AOC, total antioxidant capacity; SOD, superoxide dismutase; GPX, glutathione peroxidase; MDA, malondialdehyde; LPO, lipid peroxidation; 8-OHdG, 8-hydroxydeoxyguanosine.

1) NOR, normal breast; WB, wooden breast. Data are the mean of twelve replicates (n = 12).

*,** Show significant difference (p<0.05) and highly significant difference (p<0.01) compared with the NOR group respectively.
