# Supplementary material for: The impaired redox status and activated nuclear factor-erythroid 2-related factor 2/antioxidant response element pathway in wooden breast myopathy in broiler chickens
Source: Anim Biosci. 2020 Apr 13;34(4):652–61. doi: 10.5713/ajas.19.0953 (PMC7961296; doi:10.5713/ajas.19.0953)
Supplement: Supplementary file 1 [file ajas-19-0953-suppl.pdf]

The impaired redox status and activated Nrf2/ARE pathway in wooden breast myopathy

Xiaona Pan, Lin Zhang, Tong Xing, Jiaolong Li and Feng Gao*

Supplementary Table S1 The composition and nutrient levels of the basal diets.

**Items**	**1 to 21 d**	**22 to 42 d**
Ingredients (%)		
Corn	57.61	62.27
Soybean meal	31.00	23.00
Corn gluten meal1	3.29	6.00
Soybean oil	3.11	4.00
Limestone	1.20	1.20
Dicalcium phosphate	2.00	2.00
L-Lysine	0.34	0.35
DL-Methionine	0.15	0.08
Salt	0.30	0.30
Premix[1]	1.00	1.00
Calculated nutrient levels		
ME (MJ/kg)[2]	12.56	13.19
Crude protein (%)[3]	21.10	19.60
Calcium (%)	1.00	0.95
Available phosphorus (%)	0.46	0.39
Lysine (%)	1.20	1.05
Methionine (%)	0.50	0.42
Methionine + cysteine (%)	0.85	0.76

* and ** show significant difference ($P < 0.05$) and highly significant difference ($P < 0.01$) compared with the NOR group respectively.

[1] Premix provided per kilogram of diet: retinyl acetate for vitamin A, 12000 IU; cholecalciferol for vitamin D3, 2500 IU; DL-α-tocopheryl acetate for vitamin E, 20 IU; menadione sodium bisulphate, 1.3

mg; thiamin, 2.2 mg; riboflavin, 8.0 mg; nicotinamide, 40 mg; choline chloride, 400 mg; calcium pantothenate, 10 mg; pyridoxine HCl, 4 mg; biotin, 0.04 mg; folic acid, 1 mg; vitamin B12 (cobalamin), 0.013 mg; Fe (from ferrous sulfate), 80 mg; Cu (from copper sulphate), 8.0 mg; Mn (from manganese sulphate), 110 mg; Zn (from zinc sulfate), 60 mg; I (from calcium iodate), 1.1 mg; Se (from sodium selenite), 0.3 mg

[2] ME = metabolizable energy.

[3] 1Crude protein content was 60%.
